# Tea Polyphenol–Zinc Nanocomplexes Alleviate Diquat-Induced Liver and Small Intestine Oxidative Stress in C57BL/6 Mice

**DOI:** 10.3390/nano15171313

**Published:** 2025-08-26

**Authors:** Tingting Liu, Yang Zhao, Jie Feng

**Affiliations:** Key Laboratory of Animal Feed and Nutrition of Zhejiang Province, College of Animal Sciences, Zhejiang University, Hangzhou 310058, China; 22417001@zju.edu.cn (T.L.); zhaoyang112@zju.edu.cn (Y.Z.)

**Keywords:** Tea Polyphenol–Zinc, diquat, oxidative stress, mice, liver, small intestine, metal phenolic network

## Abstract

Oxidative stress is the key contributor to the onset of numerous diseases. Herein, we develop tea polyphenol–zinc (Tp-Zn) using a metal–polyphenol coordination strategy through a simple hybrid approach. The product is characterized by methods such as dynamic light scattering (DLS), ultraviolet–visible spectroscopy (UV–vis) and transmission electron microscopy (TEM) to evaluate the particle size and potential of Tp-Zn. Oxidative stress was induced in mice by administering diquat (25 mg/kg body weight) followed by pre-treatment with 210 mg/kg body weight tea polyphenols (TPs), 280 mg/kg body weight Tp-Zn, and 70 mg/kg body weight ZnSO_4_ for 7 days. Results showed that Tp-Zn treatment significantly improved intestinal barrier function by preventing the diquat-induced down-regulation of tight junction proteins Zonula Occludens protein 1 (ZO-1) and occludin. It also mitigated liver inflammation and damage, as evidenced by reduced serum levels of Aspartate aminotransferase (AST), Alanine aminotransferase (ALT), and Alkaline phosphatase (AKP). Furthermore, Tp-Zn enhanced the antioxidant response in both the intestine and liver by up-regulating the mRNA expression of antioxidant enzymes and reducing the levels of Malondialdehyde (MDA) and reactive oxygen species (ROS) compared to the diquat group (DIQ group). Also, the detection of ROS in the small intestine confirmed Tp-Zn markedly increased intestinal Nuclear factor erythroid 2-related factor 2 (Nrf2) expression compared to the control group. This study aims to clarify that metal–polyphenol coordination with multifaceted regulation of the inflammatory microenvironment could be a novel approach for preventing or treating oxidative stress-related diseases.

## 1. Introduction

Oxidative stress response is a ubiquitous physiological response elicited when the threat to the homeostasis is perceived by the organism due to environmental, physical, or psychological stimuli [1]. Oxidative stress can trigger the production of free radicals, such as ROS, and excessive ROS can pose a threat to animal or human health [2]. Excessive ROS can cause liver cell membrane injury and dysfunction and destruction of enzyme activity and protein structure, and by affecting liver metabolism and function, promote the release of proinflammatory factors, resulting in chronic hepatitis and fibrosis [3]. In the intestine, excessive ROS can damage the intestinal epithelial barrier, induce apoptosis of intestinal epithelial cells, change the intestinal microbiota, and activate intestinal inflammatory pathways [4,5].

Tea polyphenols form a diverse family of polyphenols in tea extracts that have excellent antioxidant properties, especially in preventing the free radical chain reaction caused by ROS [6]. Recently, studies reported that several natural plant extracts have an ability to reduce free radical production by chelating metal ions, thereby supporting the maintenance of redox homeostasis in animals [7,8,9,10,11,12,13], and some metal–organic complexes have been developed and proven effective in the treatment of various diseases [7,8,14,15,16,17,18,19]. Curcumin and zinc complex significantly reduces inflammatory responses and oxidative stress in diabetic wound healing [13]. Composite nanoparticles of iron and curcumin exhibit significant antioxidant and anti-inflammatory activities, enabling effective treatment of osteoarthritis [17]. Studies in vitro have shown that Tp-Zn scavenges OH^•^, O_2_^•−^, and DPPH more efficiently than TP, suggesting that specific tea polyphenols such as epigallocatechin gallate (EGCG) and catechins chelate with zinc to exert stronger free radical scavenging ability than free tea polyphenols alone [20], while there are few studies on the antioxidant effects of Tp-Zn in vivo. Current research primarily focuses on the individual effects of TP and zinc, with limited studies on the synergistic antioxidant effects of their complexes. Moreover, most existing studies employ in vitro cell models, lacking validation in in vivo models. Particularly in diquat-induced models, the antioxidant effects of Tp-Zn have not been adequately investigated.

To investigate the antioxidant function of Tp-Zn in vivo, we hypothesize that Tp-Zn can effectively alleviate the oxidative stress damage in the liver and small intestine of mice induced by diquat. This study utilized a diquat-induced oxidative stress model to explore the effect of Tp-Zn in alleviating oxidative stress state and its potential mechanism in C57BL/6 mice.

## 2. Materials and Methods

### 2.1. Synthesis of Tp-Zn

Tp-Zn nanoparticles were prepared by gently mixing TP and ZnCl_2_. Briefly, under nitrogen protection, equimolar solutions of TP (0.1 M) and ZnCl_2_ (0.1 M) were simultaneously added to deionized water with constant stirring. The reaction proceeded at room temperature for 20 min, yielding a milky white suspension. The product was purified by differential centrifugation (3000× *g*, 15 min), washed three times with absolute ethanol, and lyophilized to obtain the Tp-Zn. The TP used in our study was purchased from Sigma-Aldrich, Darmstadt, Germany (Product No. P1204). The zinc chloride used was sourced from Aladdin, Shanghai, China (Product No. Z112526).

### 2.2. Structural Characterization of Tp-Zn

#### 2.2.1. UV-Vis Spectroscopy Analysis

During the preparation of Tp-Zn, 5 mL of Tp-Zn solution was measured. Scanning and recording spectra were in the range of 200–500 nm by a UV-2600 spectrophotometer (Techcomp Limited, Hong Kong, China).

#### 2.2.2. Fourier-Transform Infrared Spectroscopy (FT-IR) Analysis

Tp-Zn sample powder and KBr were dried at 110 °C to constant weight. The sample was made into transparent and uniform tableting by the KBr tableting method, and FT-IR spectra were analyzed by an IS50 spectrometer (Thermo Fisher Scientific, Waltham, MA, USA) in the range of 500–4000 cm^−1^.

#### 2.2.3. Scanning Electron Microscope (SEM) Analysis and Energy-Dispersive X-Ray Spectroscopy (EDS) Analysis

Tp-Zn was deposited on a sample table and then analyzed by a field emission scanning electron microscope (SU-8010 Hitachi, Tokyo, Japan) under 3.0 kV accelerated voltage and high vacuum.

#### 2.2.4. TEM Analysis

Tp-Zn was observed at 100 keV using a transmission electron microscope analyzer (H-7650 Hitachi, Tokyo, Japan).

#### 2.2.5. Thermogravimetry (TGA) Analysis

TGA-DSC (TA Instruments Ltd., Q600, New Castle, DE, USA) was used for thermogravimetric analysis. The temperature was increased from room temperature to 800 °C, and the heating rate was 10 °C/min.

#### 2.2.6. Flame Atomic Absorption Spectroscopy

Sample solutions were prepared. The original solution was diluted 250 times and then the Zn content was detected in an atomic absorption spectrometer. The detection results are shown in Table 1.

### 2.3. Animal Experiments and Sample Collection

**Figure 1:**
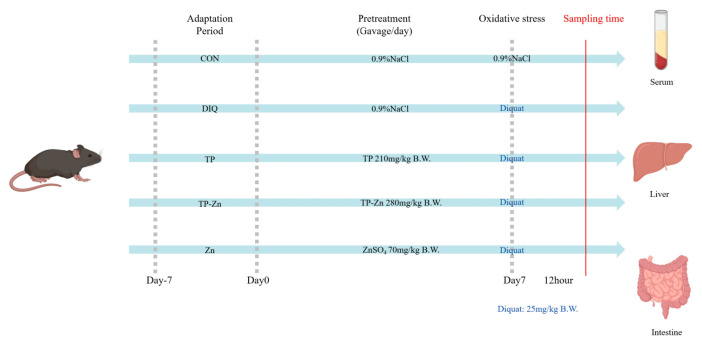
Experimental scheme for assigning mice into 5 groups (*n* = 6).

Thirty C57BL/6 male mice, 6 weeks old, were purchased from Slac Laboratory Animal Co., Ltd. (Shanghai, China). In this study, we used only male C57BL/6 mice to simplify the experimental design and minimize the potential impact of gender differences on experimental results. As shown in Figure 1, the mice were randomly divided into 5 groups (*n* = 6 per group): control group, DIQ group, TP group, Zn group, and Tp-Zn group. On day 0, by oral gavage, control group and DIQ group were given 0.9% NaCl, and the latter three groups were given 210 mg/kg of TP, 280 mg/kg of Tp-Zn, and 70 mg/kg of Zn (added as zinc sulfate), respectively, at 9:00 a.m. for seven days (Days 0–7). On Day 7, the mice in the control group were administrated saline by intraperitoneal injection; meanwhile, other groups were injected 25 mg/kg B.W. diquat (Shanghai, China). The dose of diquat was selected based on previous studies [21,22,23]. After the diquat treatments for 12 h, the experimental mice were anesthetized with Halothane, and blood was quickly collected by extracting the eyeballs. During the experiment, the mice were fed with a basal diet and kept in 12 h light/dark cycle at 20–26 °C. The serum samples were separated by centrifugation at 3000 rpm for 10 min at 4 °C. Meanwhile, the abdominal cavity was opened, and tissues were quickly collected, frozen in liquid nitrogen, and stored at −80 °C. The dose of TP was selected based on a previous study [24]. We optimized the combined dose to maximize its synergistic antioxidant effects. All animal studies were conducted in accordance with the guidelines of the Animal Ethics Committee of Zhejiang University (ZJU20240808). As stipulated by the animal welfare protocol, all efforts were made to minimize animal suffering and to use the minimum number of animals necessary to produce reliable scientific data.

#### 2.3.1. Detection of Serum Indexes

The levels of ALT and AST, the activity of AKP and superoxide dismutase (SOD), the total antioxidant capacity (T-AOC), and the levels of tumor necrosis factor-α (TNF-α) in serum of mice were detected by the assay kit according to the corresponding protocols, respectively. The assay kits were procured from Nanjing Institute of Biological Engineering (Nanjing, China).

#### 2.3.2. Detection of Liver and Small Intestine Antioxidant Index

Tissue samples were made into a 10% homogenate and centrifuged at 12,000 rpm for 5 min at 4 °C to obtain the supernatant for testing. The MDA, catalase (CAT), SOD, and T-AOC assay kits, along with the NAD(P)H dehydrogenase [quinone]1 (NQO1) and heme oxygenase-1 (HO-1) ELISA kits, were obtained from the Nanjing Institute of Biological Engineering (Nanjing, China).

#### 2.3.3. Hepatic Morphology

Dehydration, fixation, paraffin embedding, and hematoxylin and eosin (H&E) staining were performed on hepatic and intestinal samples preserved in 4% paraformaldehyde. Eventually, the hepatic sections were observed under DM300 upright microscope (Leica, Wetzlar, Germany).

#### 2.3.4. Detection of ROS Level in Liver and Small Intestine

Frozen sections were prepared from fresh liver and small intestine. Frozen slides were restored to room temperature and obvious liquid eliminated. The objective tissue was marked with a liquid blocker pen. Spontaneous fluorescence quenching reagent was added, and the section was incubated for 5 min, and then washed in running tap water for 10 min. ROS staining solution was added to the marked area and incubated at 37 °C for 30 min in a dark place. The section was incubated with 4′,6-diamidino-2-phenylindole (DAPI) solution for 10 min in a dark place at room temperature. Then, in a Rocker device, the section was washed three times with phosphate-buffered saline (PBS) (pH 7.4) for 5 min each. Residual liquid was removed; then, a coverslip was applied with anti-fade mounting medium. Finally, the sections were observed under a fluorescence microscope (Axio Imager.M2, Zeiss, Oberkochen, Germany).

#### 2.3.5. Apoptosis Assay

Following the instructions of the TdT-mediated dUTP Nick-End Labeling (TUNEL) peroxidase apoptosis detection kit (Roche, Shanghai, China), the embedded completed intestinal sections were de-stained and rehydrated. The sections were stained and reacted with fluorescein labeling solution in a moist dark environment. After rinsing three times with PBS, 3,3′-Diaminobenzidine (DAB) was added as substrate and reacted for 10 min and washed again. The processed materials were placed under a light microscope for identification.

#### 2.3.6. Quantification of Intestinal Relative mRNA Expression of Mice

Occludin, ZO-1, and Claudin mRNA expressions in small intestine tissue were measured. Total RNA from the small intestine tissue was extracted using the RNA pure Total RNA kit according to the protocol and reverse-transcribed into cDNA using a PrimeScript™ RT reagent Kit with gDNA Eraser RR047A (Sevier Biotechnology Co., LTD., Wuhan, China). Fluorescent quantitative PCR was performed using the Fluorescent quantitative PCR instrument (Applied Biosystems, Foster City, CA, USA). The data were analyzed following the 2−∆∆Ct method and calculated using β-actin as the normalization control. Primers Table 2 were synthesized by Sevier Biotechnology Co., LTD (Wuhan, China).

#### 2.3.7. Western Blot

The tissues were powdered and homogenized using Radio Immunoprecipitation Assay (RIPA) buffer with 1% Phenylmethanesulfonyl fluoride (PMSF) and phosphatase inhibitor cocktail to extract total protein. RIPA, PMSF, phosphatase inhibitor cocktail, and 5XLoading Buffer were procured from Sevier Biotechnology Co., LTD (Wuhan, China). The concentration of proteins was determined by using a BCA assay (KeyGen Biotech, Nanjing, China). Tissue protein (30 µg/lane) was electrophoresed through SDS-PAGE at 90 V for 15 min and 120 V for 1 h, and then electronically transferred to PVDF membranes at 260 mA for 1 h. After blocking with 5% de-fat milk for 2 h, the blots were incubated at 4 °C overnight with primary antibodies: Nrf2 and Kelch-like ECH-associate protein 1 (Keap1) (Proteintech, Wuhan, China). After washing with Tris-Buffered Saline with Tween-20 (TBST) buffer three times, the Horseradish Peroxidase (HRP)-conjugated secondary antibodies were applied for 2 h at room temperature. Washing was performed again, and then the chemiluminescence signals were detected by using an Electrochemiluminescence (ECL) System (Marine Engineering Bioengineering, Shanghai, China) and visualized by using a Chemiluminescence imager (Bio-Rad, Hercules, CA, USA). Finally, the images were analyzed using Image Lab software (Version 6.1).

### 2.4. Statistical Analysis

All data were reported as mean values with their SD. Data were analyzed using one-way ANOVA; *p* < 0.05 was considered to be statistically significant. All statistical analyses were performed using SPSS (Version 26.0). GraphPad Prism (Version 9.0) was used for the graphical representations.

## 3. Results

### 3.1. Synthesis and Characterization of Tp-Zn

**Figure 2:**
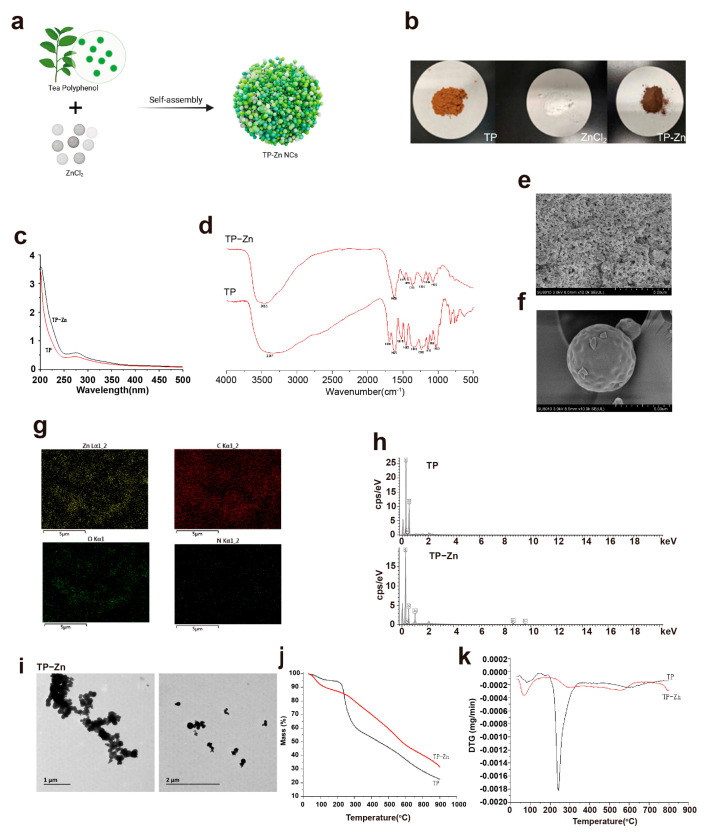
Synthesis and characterization of Tp–Zn. (**a**) Synthesis of Tp-Zn. (**b**) Comparison of TP, ZnCl_2_, and Tp–Zn. (**c**) UV–vis spectra of TP and Tp–Zn. (**d**) FT–IR spectra of TP and Tp–Zn. (**e**,**f**) SEM images; scale bar = 5 μm. (**g**,**h**) EDS of TP and Tp–Zn. (**i**) TEM images; scale bar = 1–2 μm. (**j**,**k**) TGA of TP and Tp–Zn.

Herein, ZnCl_2_ was mixed with tea polyphenols solution at room temperature to obtain Tp-Zn (Figure 2a). Comparing the appearance of tea polyphenol, zinc chloride, and Tp-Zn, Tp-Zn was quite different to TP (Figure 2b). In order to further explore the synthesis of Tp-Zn, UV-vis spectrophotometry was used to measure the ultraviolet absorption spectra of TP and ZnCl_2_ used for association and the solution obtained after association. As shown in Figure 2c, TP shows a characteristic peak of flavanol compounds at around 273 nm. After complexation with Zn^2+^ without characteristic peaks, the characteristic peak is significantly weakened. This may be due to the π → π* and p–π conjugate electron transition in the benzene ring of TP, proving the successful association of Tp-Zn. The composition and structure of Tp-Zn were characterized by FT-IR (Figure 2d). The absorption peak below 1000 cm ± 1, which is attributed to the stretching vibration of the Zn-O bond, shows a shift after the formation of the nanocomplexes. The tensile vibration peak of the carbonyl group (C=O) disappeared, which further demonstrates the successful complexation of zinc with TP. According to the results of SEM, both TP and Tp-Zn are spherical in shape (Figure 2e,f). However, the molecular particle size of Tp-Zn was significantly smaller than that of TP, which may facilitate the absorption and utilization in the animal intestine. Figure 2g shows the results of EDS mapping. It can be seen that the elements C, O, and Zn are evenly distributed in the particles. According to EDS statistics, the proportion of Zn element is 23 ± 0.75% (Figure 2h). The TEM image in Figure 2i more intuitively observes the morphology of Tp-Zn. It can be observed that Tp-Zn presents a relatively uniform spherical structure with a particle size of about less than 1 μm. The thermodynamic properties of Tp-Zn were analyzed by TGA (Figure 2j,k). In an oxygen atmosphere, both TP and Tp-Zn produce continuous weight loss. In the range of 238 °C to 800 °C, the loss rate of Tp-Zn was lower than TP with increasing temperature, indicating that Tp-Zn is more thermally stable than TP. It is calculated that the content of Zn^2+^ should be about 24%, which is basically consistent with the calculation result of EDS. Atomic absorption spectrometry (AAS) was used to determine the content of zinc in Tp-Zn. As shown in Table 2, we can calculate the concentration of zinc in the solution of Tp-Zn to be 227.53 mg/L. Zinc is about 11.38 mg in the 50 mL Tp-Zn solution, and TP is about 38.62 mg. Therefore, the mass ratio of TP to zinc in Tp-Zn is about 3:1, which is basically consistent with the calculation result of TGA and EDS. In summary, these results demonstrate the successful synthesis of Tp-Zn through the coordination of zinc with TP and good stability.

### 3.2. Serum Index

**Figure 3:**
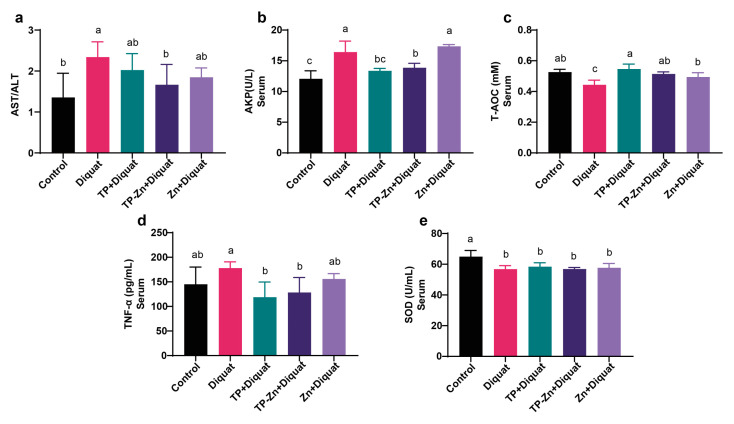
Tp-Zn ameliorates DIQ-induced oxidative stress exacerbation in serum in mice (*n* = 6). (**a**) AST/ALT in serum (*n* = 6). (**b**) AKP in serum (*n* = 6). (**c**) T-AOC in serum (*n* = 6). (**d**) TNF-α in serum (*n* = 6). (**e**) SOD in serum (*n* = 6). abc: Means without a common letter differ significantly (*p* < 0.05). Error bars denote SD.

As shown in Figure 3a–e, compared with the control group, the levels of AST/ALT and AKP in the serum of the DIQ group were significantly increased (*p* < 0.05), and the level of T-AOC was significantly decreased (*p* < 0.05). Compared with the DIQ group, the levels of AKP and TNF-α in the TP group were significantly decreased (*p* < 0.05), and the levels of AST/ALT, AKP, and TNF-α in the Tp-Zn group were significantly decreased (*p* < 0.05). The data on mouse weight and food intake are provided in the Supplementary Materials Figure S1.

### 3.3. Tp-Zn Prevents Hepatic Injury in Diquat-Induced Mice

**Figure 4:**
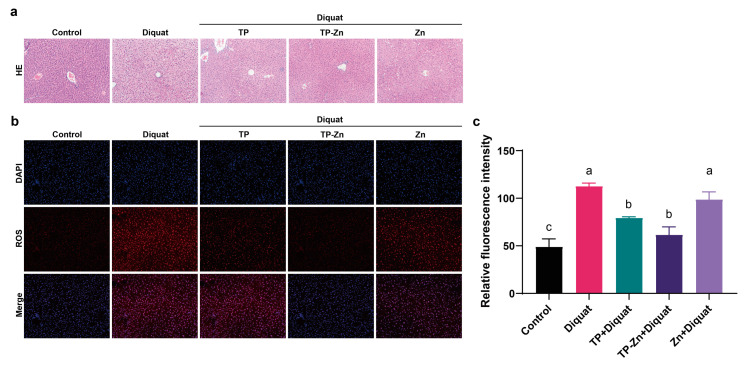
Effects of TP, Tp-Zn, and Zn pre-treatment on liver function in mice. (**a**) H&E staining of liver (100×). (**b**) Immunofluorescence staining of liver tissues from experimental mice. (**c**) Fluorescence intensity of ROS in liver. abc: Means without a common letter differ significantly (*p* < 0.05). Error bars denote SD.

As shown in Figure 4a, compared to the control group, liver cells of mice in the DIQ group showed obvious shrinkage and vacuoles existed, indicating that diquat caused substantial damage to the liver of mice. A few vacuoles were also present in the TP and Zn groups, but no obvious lesions were observed in the liver sections of the Tp-Zn group compared to the DIQ group, thus indicating that Tp-Zn could alleviate liver damage in mice caused by diquat. Compared with the control group, diquat significantly increased the level of ROS, which corresponds to the results of H&E staining of liver. Compared with the DIQ group, Tp-Zn, TP, and zinc treatment alleviated the damage caused by diquat and reduced the ROS content in the liver (Figure 4b,c); the Tp-Zn group showed the best protection against liver damage in mice.

### 3.4. Liver Antioxidant Index

**Figure 5:**
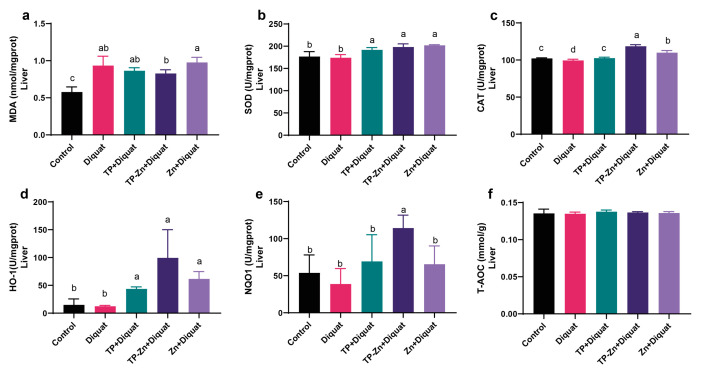
Effect of Tp-Zn on the antioxidant ability of the liver in diquat-induced mice (*n* = 6). (**a**–**f**) Levels of MDA, SOD, CAT, HO-1, NQO1, and T-AOC in the liver of different groups of mice. abcd: Means without a common letter differ significantly (*p* < 0.05). Error bars denote SD.

As shown in Figure 5a–f, compared with the control group, the levels of MDA in the liver of the DIQ group were significantly increased (*p* < 0.05). Compared with the DIQ group, Tp-Zn group significantly increased the levels of SOD, CAT, HO-1, and NQO1 (*p* < 0.05), which showed superior efficacy to the TP group.

### 3.5. Tp-Zn Prevents Intestinal Injury and Apoptosis in Diquat-Induced Mice

**Figure 6:**
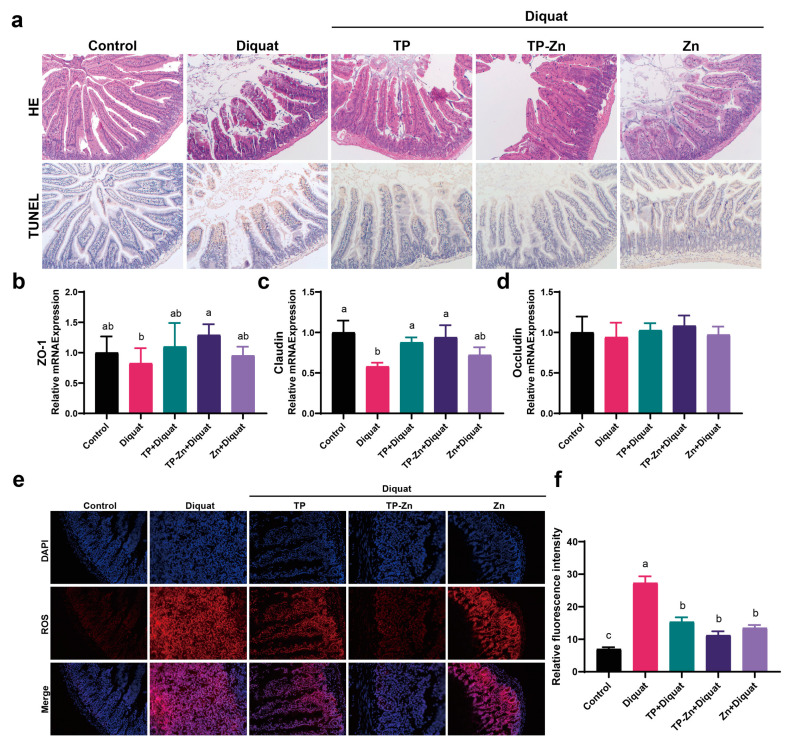
Effects of TP, Tp-Zn, and Zn pre-treatment on intestinal barrier in diquat-induced mice. (**a**) H&E staining of the small intestine (100×) and apoptosis of small intestine epithelial cells (100×). (**b**–**d**) The relative mRNA expression levels of intestinal tight junction-related genes in intestine (*n* = 6). (**e**) Immunofluorescence staining of intestine tissues from experimental mice. (**f**) Fluorescence intensity of ROS in the small intestine. abc: Means without a common letter differ significantly (*p* < 0.05). Error bars denote SD.

The intestinal revealed that DIQ significantly disrupted the structure of intestinal villi, while the other three treatments showed varying degrees of improvement (Figure 6a). TUNEL showed that the intestinal villi apical epithelial cells in the DIQ group were seriously apoptotic (Figure 6a). Compared to the DIQ group, treatment of TP and Tp-Zn alleviated the apoptosis of intestinal epithelial cells and the destruction of intestinal villi structure, but the degree of apoptosis was still higher than that in the control group. The mRNA expression of tight junction proteins in mice intestine were measured to further evaluate the integrity of the intestinal barrier (Figure 6b–d). Compared with the control group, diquat significantly down-regulated the mRNA expression of tight junction proteins ZO-1 and Claudin (*p* < 0.05), while Tp-Zn effectively alleviated the decrease in mRNA expression of these two proteins, with a better effect than TP and Zn. Tp-Zn, TP, and Zn treatment alleviated intestinal damage caused by diquat and reduced the ROS content in the small intestine compared with the DIQ group (Figure 6e,f). The results showed that pre-treatment with Tp-Zn effectively alleviated the oxidative stress injury of small intestine induced by diquat, and the effect was more effective than that of TP group and Zn group.

### 3.6. Small Intestine Antioxidant Index

**Figure 7:**
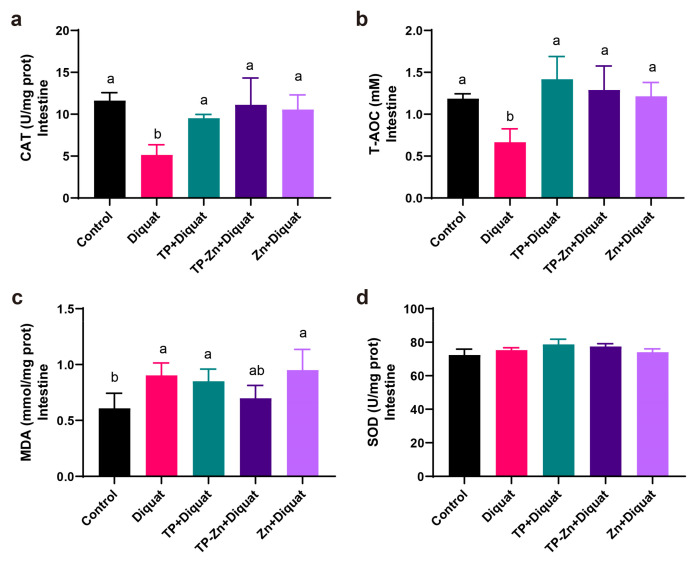
Effect of Tp-Zn on the antioxidant ability of the intestine in diquat-induced mice (*n* = 6). (**a**–**d**) Levels of CAT, T-AOC, MDA, SOD in the intestine of different groups of mice. ab: Means without a common letter differ significantly (*p* < 0.05). Error bars denote SD.

As shown in Figure 7a–d, compared with the control group, the levels of CAT and T-AOC in the intestine of the DIQ group were significantly decreased (*p* < 0.05), and the level of MDA increased (*p* < 0.05). Compared with the DIQ group, the levels of CAT and T-AOC in the TP group and Tp-Zn group were significantly increased (*p* < 0.05), while the value of the Tp-Zn group was closer to the control group.

### 3.7. Tp-Zn Alleviates Diquat-Induced Intestinal Oxidative Stress: Potential Involvement of the Nrf2-Mediated Signaling Pathway

**Figure 8:**
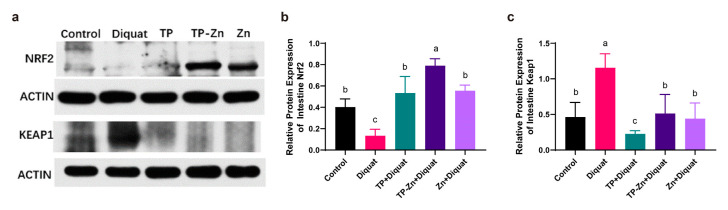
Expression of antioxidant related proteins in small intestine (*n* = 5). (**a**) The expression levels of Nrf2 signaling pathway (Nrf2 and Keap1). (**b**,**c**) Quantitative analysis of the protein expression levels of Nrf2 and Keap1. abc: Means without a common letter differ significantly (*p* < 0.05). Error bars denote SD.

The Nrf2-ARE signaling pathway is a critical mechanism for regulating antioxidant responses in organisms. As shown in Figure 8a–c, our results demonstrated that the DIQ group exhibited a significant reduction in Nrf2 protein expression (*p* < 0.05) and a significant increase in Keap1 protein levels (*p* < 0.05) compared to the control group. In contrast, the Tp-Zn group displayed a markedly higher Nrf2 protein expression than the DIQ group (*p* < 0.01). Additionally, compared to the TP group, the Tp-Zn group significantly upregulated the expression of both Nrf2 and Keap1 proteins. These findings suggested that Tp-Zn may activate the Nrf2 signaling pathway and subsequently induce a cascade of antioxidant response elements, thereby mitigating diquat-induced oxidative stress in mice.

## 4. Discussion

Oxidative stress can trigger the production of free radicals, such as ROS, and excessive ROS can pose a threat to animal or human health. Tp-Zn has strong scavenging ability against oxygen free radicals and antioxidant activity in vitro [25]. Studies have shown that diquat, a strong oxidant, can induce oxidative stress in animals [26,27]. Thus, our study used diquat to build an oxidative stress model and investigated the effects and mechanisms of Tp-Zn in alleviating oxidative stress and hepatic and intestinal injury.

Our results revealed that compared with the control group, diquat treatment significantly increased the serum levels of AKP, TNF-α, and AST/ALT and decreased the levels of SOD and T-AOC. Compared with the DIQ group, Tp-Zn significantly reduced the serum levels of AKP, AST/ALT, and TNF-α, and the T-AOC level was significantly increased. The abnormal increase in AST/ALT ratio and AKP values are usually considered that liver cells have been seriously damaged [28]. TNF-α is a cytokine produced by immune cells that promotes inflammation [29,30]. These results indicated that diquat reduced the serum antioxidant level and increased the content of inflammatory factors in the serum of mice, while Tp-Zn significantly increased serum antioxidant levels and reduced liver damage in mice.

The current results show that diquat causes liver damage in mice, leading to liver cell collapse, significantly increasing the levels of MDA and ROS, and significantly decreasing CAT levels. Tp-Zn effectively increased the levels of CAT, NQO1, HO-1, and SOD in the liver of mice and significantly reduced the level of ROS, which alleviated the liver damage induced by diquat in mice. Feng et al. found that quercetin and tea polyphenols significantly inhibited HNE-induced ROS production and cytotoxicity in rat liver epithelial RL34 cells [31]. Both NQO1 and HO-1 protect the body by reducing the production of free radicals in the body [32]. SOD can react with O_2_^•−^ to form H_2_O_2_, which is then further degraded into water by CAT and peroxidase [33]. Compared with TP, the protective effect of Tp-Zn on the liver is more obvious, which may be due to the zinc in Tp-Zn strengthening the activity of the phenolic hydroxyl group, causing Tp-Zn to have stronger antioxidant activity. Tea polyphenols include flavonoids, catechins, anthocyanins, and phenolic acids [25]. The reason why the complex of metal elements and flavonoids can enhance the antioxidant activity of flavonoids may be due to the formation of additional superoxide dismutable metal centers [34]. For instance, certain polyphenols within TPs, such as epigallocatechin gallate (EGCG) or catechins, are known to interact with zinc ions, potentially forming complexes that exhibit stronger antioxidant activity compared to free TPs. Further research is needed to precisely identify and characterize these specific complexes and their contributions to the overall antioxidant effects.

The intestinal barrier, which consists of a mucus layer and a single layer of epithelial cells interconnected by tight junctions (TJs), is essential for the maintenance of homeostasis in the intestine [35]. The current study showed that compared with the control group, diquat treatment caused intestinal injury, destroyed the villous morphology of the small intestine, promoted the apoptosis of jejunal epithelial cells, increased the contents of MDA and ROS, decreased the levels of CAT and T-AOC, damaged the intestinal barrier, and decreased the expression of antioxidant protein Nrf2 in mice. Diquat has the same effect on other animals [26,36,37,38,39]. Bártíková et al. found that oral administration of low-dose green tea extract had the positive effect of improving oxidative stress in mice intestines [40]. Our results also found that TP can restore the passive effect of diquat, and Tp-Zn showed better effect. Compared with the DIQ group, Tp-Zn significantly increased CAT and T-AOC levels compared to the TP group, and the MDA level of the Tp-Zn group was restored to the level of the control group, while the MDA level of the TP group was significantly higher than that of the control group. Comparing the effects of TP with Tp-Zn, Tp-Zn tended to be superior to TP in most of the parameters. A previous study mainly showed the scavenging ability of Tp-Zn on DPPH, O_2_^•−^, and OH^•^ in vitro, indicating that the scavenging ability of Tp-Zn on free radicals was significantly higher than that of TP [25]. Tea polyphenols, known for their antioxidant properties, may include specific polyphenolic compounds that can chelate with zinc ions to form stable complexes. This chelation likely enhances the antioxidant capacity of the combined system. For instance, certain polyphenols within TPs, such as epigallocatechin gallate (EGCG) or catechins, are known to interact with zinc ions, potentially forming complexes that exhibit stronger antioxidant activity compared to free TPs. Further research is needed to precisely identify and characterize these specific complexes and their contributions to the overall antioxidant effects.

This study aims to investigate the protective effects of Tp-Zn against oxidative stress damage and its underlying mechanisms. According to previous studies, TP can ameliorate streptozotocin (STZ)-induced diabetic mice and oxidative damage by activating Keap1/Nrf2/ARE Signaling pathways [41]. The Nrf2 signaling pathway is one of the main pathways for antioxidant functions in animals. Nrf2, as a key sensor and transcription factor of the antioxidant system, regulates the expression of various antioxidant enzymes, such as SOD, CAT, and glutathione peroxidase (GPx) [42]. The change of Nrf2 content represents the activation of the NRF2-ARE signaling pathway [43]. Therefore, in this study, we detected the expression levels of Nrf2 and its downstream genes. The results showed that Tp-Zn supplementation significantly increased Nrf2 expression and decreased KEAP1 expression after diquat stimulation, indicating that Tp-Zn can more effectively activate the Nrf2 pathway to exert its antioxidant effects. However, the current study only observed changes in the expression levels of Nrf2 and some of its downstream genes. The specific mechanisms by which Tp-Zn activates the Nrf2 pathway remain unclear. Future research should further explore the regulatory effects of Tp-Zn on other key molecules in the Nrf2 pathway and whether it interacts directly with Nrf2 to regulate the expression of downstream antioxidant enzymes, thereby elucidating the antioxidant mechanisms of Tp-Zn more comprehensively.

This study is the first to explore the regulatory effects of Tp-Zn on the Nrf2 pathway and its protective effects against oxidative stress damage, providing a new direction for the potential applications of Tp-Zn. By setting up different treatment and control groups, this study systematically evaluated the effects of Tp-Zn on the expression of genes related to the Nrf2 pathway. Although this study found that Tp-Zn can activate the Nrf2 pathway, the specific targets and signaling transduction pathways involved remain unclear. Future research needs to further explore the interactions between Tp-Zn and key molecules in the Nrf2 pathway, as well as whether it involves the synergistic regulation of other signaling pathways. Additionally, this study only observed the effects of short-term treatment with Tp-Zn. The long-term effects of Tp-Zn supplementation on antioxidant capacity and potential adverse reactions are still unknown. Long-term effect studies are crucial for evaluating the safety and effectiveness of Tp-Zn as a potential therapeutic agent. Future studies should further validate the antioxidant effects of Tp-Zn and its regulatory effects on the Nrf2 pathway in animal models. Future research should delve into the regulatory mechanisms of Tp-Zn on key molecules in the Nrf2 pathway, including its direct interactions with Nrf2, its effects on Nrf2 transcriptional activity, and the fine regulation of downstream antioxidant enzyme expression. Additionally, the synergistic interactions between Tp-Zn and other signaling pathways should be explored to provide a comprehensive understanding of its antioxidant mechanisms.

In summary, our study has preliminarily revealed the potential mechanisms by which Tp-Zn exerts antioxidant effects through the activation of the Nrf2 pathway, laying the foundation for further research and application of Tp-Zn. However, this study still has certain limitations. Future research needs to further validate its antioxidant effects, explore its mechanisms in depth, and assess its long-term effects and clinical application potential.

## 5. Conclusions

Herein, Tp-Zn with good biosafety and biocompatibility have been successfully prepared through a simple hybrid approach. In conclusion, our study demonstrated that prophylactic Tp-Zn treatment effectively alleviated diquat-induced hepatic and intestinal injury in mice, and Tp-Zn may be a potential nutraceutical for preventing or treating oxidative stress-related diseases.

## Figures and Tables

**Table 1:** The content of zinc in Tp–Zn.

	Blank	c(Tp-Zn) (250×)	c(Zn^2+^) (mg/L)
1	0.0102	0.4185	0.9085
2	0.0100	0.4184	0.9083
3	0.0105	0.4205	0.9129
Average	0.0102	0.4191	0.9101

**Table 2:** Primers of target genes.

Gene	Primer Sequences(5′-3′)	Primer Length (bp)
Claudin	GAAAAATGGACGAACTGGGCTCC CCAGAACGGAGGCAGCAATCAT	150
Occludin	TGGCAAGCGATCATACCCAGAG CTGCCTGAAGTCATCCACACTC	103
ZO-1	GTTGGTACGGTGCCCTGAAAGA GCTGACAGGTAGGACAGACGAT	133
Actin	CATTGCTGACAGGATGCAGAAGG TGCTGGAAGGTGGACAGTGAGG	138

## Data Availability

All data generated or analyzed during this study are available from the corresponding author upon reasonable request.

## Supplementary Materials

The following supporting information can be downloaded at: https://www.mdpi.com/article/10.3390/nano15171313/s1, Figure S1: (a) Average daily feed intake (ADFI) in diquat-treated mice; (b) Changes in body weight of mice (*n* = 6).

## Abbreviations

The following abbreviations are used in this manuscript:

AKP	Alkaline Phosphatase
ALT	Alanine Aminotransferase
ARE	Antioxidant response element
AST	Aspartate aminotransferase
CAT	Catalase
DAPI	4′,6-diamidino-2-phenylindole
EDS	Energy-dispersive X-ray spectroscopy
FT-IR	Fourier-transform infrared spectroscopy
HE	Hematoxylin–Eosin staining
HO-1	Heme oxygenase 1
Keap1	Kelch-like ECH-associated protein 1
MDA	Malondialdehyde
Nrf2	Nuclear factor erythroid 2-related factor 2
NQO1	NAD(P)H dehydrogenase [quinone]1
PBS	Phosphate buffer saline
ROS	Reactive oxygen species
SEM	Scanning electron microscope
SOD	Superoxide Dismutase
T-AOC	Total antioxidant capacity
TEM	Transmission electron microscope
TGA	Thermogravimetry
TNF-α	Tumor necrosis factor-α
TP	Tea Polyphenols
Tp-Zn	Tea Polyphenol–Zinc
UV-vis	Ultraviolet–visible spectroscopy
ZO-1	Zonula Occludens protein 1
