# Supplementary material for: Tea Polyphenol–Zinc Nanocomplexes Alleviate Diquat-Induced Liver and Small Intestine Oxidative Stress in C57BL/6 Mice
Source: Nanomaterials (Basel). 2025 Aug 26;15(17):1313. doi: 10.3390/nano15171313 (PMC12430083; doi:10.3390/nano15171313)
Supplement: Supplementary file 1 [file nanomaterials-15-01313-s001.zip › nanomaterials-3785676-supplementary.pdf]

Tea Polyphenol-Zinc nanocomplexes alleviate diquat-induced liver and small intestine oxidative stress in C57BL/6 Mice

Tingting Liu, Yang Zhao, Jie Feng*

Key Laboratory of Animal Feed and Nutrition of Zhejiang Province, College of Animal Sciences, Zhejiang University, Hangzhou, 310058, China
*Correspondence: fengj@zju.edu.cn

Supporting Information

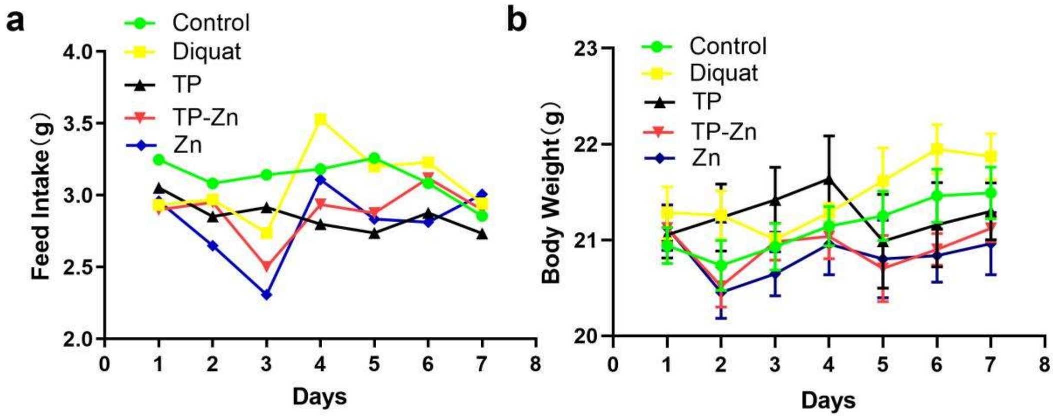

Fig.S1. Effects of TP-Zn on growth in diquat-induced mice. (a)Average daily feed intake (ADFI) in diquat-treated mice; (b) Changes in body weight of mice(n = 6).
